# Salicylate-Induced Auditory Perceptual Disorders and Plastic Changes in Nonclassical Auditory Centers in Rats

**DOI:** 10.1155/2014/658741

**Published:** 2014-05-07

**Authors:** Guang-Di Chen, Kelly E. Radziwon, Nina Kashanian, Senthilvelan Manohar, Richard Salvi

**Affiliations:** Center for Hearing and Deafness, State University of New York at Buffalo, 137 Cary Hall, 3435 Main Street, Buffalo, NY 14214, USA

## Abstract

Previous studies have shown that sodium salicylate (SS) activates not only central auditory structures, but also nonauditory regions associated with emotion and memory. To identify electrophysiological changes in the nonauditory regions, we recorded sound-evoked local field potentials and multiunit discharges from the striatum, amygdala, hippocampus, and cingulate cortex after SS-treatment. The SS-treatment produced behavioral evidence of tinnitus and hyperacusis. Physiologically, the treatment significantly enhanced sound-evoked neural activity in the striatum, amygdala, and hippocampus, but not in the cingulate. The enhanced sound evoked response could be linked to the hyperacusis-like behavior. Further analysis showed that the enhancement of sound-evoked activity occurred predominantly at the midfrequencies, likely reflecting shifts of neurons towards the midfrequency range after SS-treatment as observed in our previous studies in the auditory cortex and amygdala. The increased number of midfrequency neurons would lead to a relative higher number of total spontaneous discharges in the midfrequency region, even though the mean discharge rate of each neuron may not increase. The tonotopical overactivity in the midfrequency region in quiet may potentially lead to tonal sensation of midfrequency (the tinnitus). The neural changes in the amygdala and hippocampus may also contribute to the negative effect that patients associate with their tinnitus.

## 1. Introduction

One of the most reliable methods of inducing transient tinnitus involves administering a large dose of sodium salicylate (SS) [1, 2], the active ingredient in aspirin. Consequently, SS is often used to investigate the biological underpinning of tinnitus as well as the ensuing peripheral hearing loss that accompanies it [2–7]. The biological mechanisms underlying SS-induced hearing loss and tinnitus have been extensively studied in the classical auditory system. In the cochlea, salicylate competitively binds to prestin in outer hair cells (OHC); this attenuates OHC electromotility, distortion product otoacoustic emissions (DPOAE), and the cochlear compound action potential (CAP) and contributes to SS-induced hearing loss [2, 8, 9].* In vitro*, SS suppresses GABAergic inhibition [10–12]; these changes are believed to contribute to neural hyperactivity, changes in gain control and synaptic rescaling, and plastic reorganization in the classical auditory pathway, effects that presumably contribute to the SS-induced auditory perceptual disorders [8, 13–21].

Neural signals in the classical auditory pathway make their way to many other brain regions involved in auditory learning/memory, sound-related emotional response, vocal production, multisensory integration, and motor control [22–55]. Brain regions outside the classical auditory system are postulated to gate or modulate the severity of tinnitus and hyperacusis [56–58]. Indeed, clinical evidence suggests that the amygdala, striatum (Str), hippocampus (HC), and frontal cortex participate in tinnitus and hyperacusis [59–62]. Consistent with clinical data, we found that SS enhanced sound-evoked responses and altered the tonotopy of neurons in the lateral amygdala (LA) [56]. Previous c-fos immunolabeling studies suggested that SS could induce electrophysiological changes in several other nonauditory structures [63, 64]. To investigate the functional changes induced by SS in other nonauditory structures linked to tinnitus, we recorded from the Str, LA, HC, and cingulate (Cg) to determine how the electrophysiological properties of neurons in these structures were altered by a high dose of SS known to induced tinnitus and mild cochlear hearing loss.

## 2. Experimental Methods and Materials

### 2.1. Subjects

Forty-three Sprague-Dawley rats (3–5 months of age, Charles River Laboratories, Wilmington, MA) were housed in the Laboratory Animal Facility (LAF) at the University at Buffalo and given free access to food and water. The colony room was maintained at 22°C with a 12-hour light-dark cycle. All procedures used in this project were approved by the Institutional Animal Care and Use Committee (HER05080Y) at the University at Buffalo and carried out in accordance with NIH guidelines.

### 2.2. Salicylate Administration 

SS (Sigma-Aldrich, no. S3007) was dissolved in saline (50 mg/mL). Rats were injected with saline (5 mL/kg, i.p.) or SS (200 or 250 mg/kg, i.p.); these doses of SS have previously been shown to consistently enhance the amplitude of acoustical startle responses and induce tinnitus in rats [4].

### 2.3. Behavioral Measurement of Auditory Threshold

Five rats were trained in a go/no-go operant conditioning paradigm to detect broadband noise bursts in a sound attenuating chamber. Rats were food restricted and kept at approximately 85% of their free-feeding weight during the course of experiment. The broadband noise burst (300 ms duration, 5 ms rise/fall time, cosine gated) used in this experiment contained frequencies up to 42 kHz.

A rat began a trial by placing its nose in a nose-poke hole, which initiated a variable waiting interval ranging from 1 to 4 s. The rat had to maintain its position in the nose-poke hole until it heard a noise burst or the trial was aborted. In the* go* condition, the target stimulus was the noise burst. If the rat detected this signal, it removed its nose from the nose-poke hole resulting in a food reward (45 mg dustless rodent grain pellets, Bio-Serv); a* hit* was recorded if the rat correctly responded to the broadband noise within 2 s. A* miss* was recorded if the rat failed to remove its nose from the nose-poke within the 2 s response interval. Approximately 30% of all trials were* catch* trials. These constituted the* no-go* part of the procedure; noise bursts were not presented during these trials. If the rat removed its nose during a* catch* trial, a* false alarm* was recorded and the rat received a 4 s timeout, during which the house light was turned off and the rat could not start another trial. However, if the rat continued to nose-poke, a* correct rejection* was recorded. No reinforcement was given for a* correct rejection*.

The noise bursts were presented according to the psychophysical method of constant stimuli (MOCS). Within each 10-trial block, seven predetermined target intensities were presented randomly along with 3* catch* trials. The target intensities were chosen so that only the lowest one or two intensities were estimated to be below threshold, whereas the remaining intensities were well above threshold. Mean* hit* and* false alarm* rates were used to calculate thresholds using signal detection theory with a threshold criterion of *d*′ = 1.5.

After baseline noise-burst thresholds were collected, the rats were tested once per week with either a single i.p. injection of sodium salicylate (200 mg/kg) dissolved in saline (50 mg/mL) or an equivalent volume of saline (control). The injections were administered 2 h before testing.

### 2.4. Behavioral Measurement of Tinnitus

Three rats were trained on a two-alternative forced choice identification task designed to detect tinnitus. The material and methods for this behavioral measure are similar to those described previously [65]. Rats were food restricted to 85–90% of free feeding weight during the course of the experiment. The rats were trained to activate the left feeder trough in the presence of a steady-state narrowband noise (NBN: 1/8 octave band, center frequencies randomized across trials: 4, 5, 6, 8, or 11 kHz at 70 dB SPL) and to activate the right feeder trough in the presence of an amplitude-modulated noise (AM: broad-band noise at 70 dB SPL, 100% modulation depth at 5 kHz) or no sound (Quiet). One of the three acoustic conditions was continuously present in the chamber at the start of each trial. The rat would initiate a trial by holding its nose in the center nose-poke for a random interval ranging from 4 to 8 s. After this waiting interval, a white light above the nose-poke would illuminate, serving as a “go cue” that initiated the start of a trial. Directly after the go cue, the rats responded to the feeder associated with the acoustic condition. Correct responses were immediately rewarded with a food pellet (45 mg dustless grain pellets, Bio-Serv) delivered to the respective feeder associated with each of the three stimuli while incorrect responses were punished with a 60-second “time out” in which the rat was unable to initiate a new trial. After the rat responded to a feeder trough and received either a pellet or a time out, the acoustic condition changed and another trial began. Trial sequences were randomized using criteria outlined previously [66, 67] in order to minimize guessing and strategized behavior. Percentage of trial types was split up evenly between the two feeders (NBN at 50%; AM at 30%; Quiet at 20%). Throughout training the rate of reinforcement was progressively reduced from 100% to 70%, that is, partial reinforcement to minimize extinction of the learned behaviors. Rats were trained to a criterion of >80% correct response for each acoustic condition.

Once a rat met the criteria for at least 4 consecutive baseline days they were injected with either a 200 mg/kg (i.p.) dose of sodium salicylate dissolved in 50 mg/mL saline or an equivalent volume of saline 2 h before testing. On the tinnitus testing days with injections of either saline (control) or salicylate, Quiet trials were unreinforced, but a response to either feeder was required to complete the trial. Evidence of tinnitus was described as a shift in response on Quiet trials from the feeder previously associated with AM and Quiet trials to the feeder associated with the steady NBN trials; a shift in response preference from the Quiet feeder to the steady NBN feeder was interpreted as evidence that the rat perceived a steady state sound in the absence of any acoustic stimuli. On tinnitus testing days with either saline (control) or salicylate, there was no reinforcement for Quiet trials; however, the rate of reinforcement for AM and NBN trials was increased from 70% to 90% in order to compensate for the lack of food reinforcement on Quiet trials. If animals shifted their responses on Quiet trials when they were injected with saline, then we would assume that the animals are only sensitive to the reinforcement probabilities of the testing schedule and may not be experiencing tinnitus. However, if the animals only show a shift during Quiet trials when injected with salicylate, while maintaining accurate performance on AM and NBN trials, then we can interpret this as evidence of tinnitus.

### 2.5. Estimates of Loudness Perception Using Reaction Time Measures

Using reaction time as a surrogate of loudness perception, 7 rats were tested on a go/no-go operant conditioning paradigm to detect broadband noise bursts in quiet. The procedure for this experiment was identical to the one used to obtain broadband noise thresholds. However, the intensity of the broadband noise bursts (300 ms duration, 5 ms rise/fall time, cosine gated) in this condition ranged from 30 to 90 dB SPL instead of near-threshold levels. Reaction times measures were taken from the onset of the noise burst to the time the rat removed its nose from the nose-poke hole. Only reaction times for “hits” (when the animal correctly detected the stimulus) were included in our analysis.

As in the broadband noise threshold condition, the rats were tested once per week with either a single i.p. injection of sodium salicylate (200 mg/kg) dissolved in saline (50 mg/mL) or an equivalent volume of saline (control). The injections were administered 2 h before testing and all 7 animals received saline and salicylate injections. Three of the rats received saline injections first while the other 4 received salicylate injections first.

### 2.6. Acoustic Startle Reflex Amplitude

Six rats were tested on an acoustic startle reflex paradigm in order to assess the magnitude of the animal's reflexive motoric response to a sudden, unexpected loud sound [68]. As described in our previous publications, each rat was placed in an acoustically-transparent wire-mesh (0.5 cm × 0.5 cm) cage (20 cm × 7 cm × 6 cm) mounted on a Plexiglas base (20 cm × 10 cm) which rested on a pressure sensitive 35 mm piezoelectric transducer (MCM 28-745) that generated a voltage response proportional to the magnitude of the startle response [20, 69, 70]. Sound stimuli and startle responses were produced and measured with Tucker Davis hardware and custom software as described previously [71]. Stimuli were generated by a real-time processor (TDT RX6) with a ~100 kHz sampling rate, amplified, and delivered through a speaker (Fostex FT17H) placed approximately 25 cm above the startle platform. The startle stimulus consisted of a single broadband noise burst (20 ms duration, 0.1 nominal rise/fall time) presented at ten intensities from 70 to 115 dB SPL. Ten trials were presented in a pseudorandom order (15–25 s intertrial intervals) per intensity. Startle amplitudes for each rat were obtained following i.p. injections of either sodium salicylate (250 mg/kg) dissolved in saline (50 mg/mL) or an equivalent volume of saline (control condition). All six rats were tested with saline and SS; three rats received the saline control injection first while the other three rats received the salicylate injection first. The injections were always administered 2 h before testing.

### 2.7. Electrodes

A customized electrode assembly consisting of 2–4 polyimide-insulated tungsten electrodes (FHC Inc., impedance ~1 MΩ) or a 16-channel, linear silicon microelectrode (A-1x16–10 mm 100–177, NeuroNexus Technologies) was used to record neural activity in the LA, Str, HC, and Cg.

### 2.8. Surgery, Stimuli, and Physiological Recordings

Details of our electrophysiological techniques are described in detail in previous publications [8, 13, 56]. Briefly, rats were anesthetized with ketamine and xylazine (50 and 6 mg/kg i.m.) and placed in a stereotaxic apparatus with blunted ear bars. The dorsal surface of the skull was exposed and a head bar was firmly attached to the skull using a screw and dental cement. The head bar was attached to a rod mounted on a magnetic base. The assembly was used to hold the animal's head in the stereotaxic frame after removing the right ear bar. This allowed the right ear to be acoustically stimulated using a free-field loudspeaker. A craniotomy was performed on the skull (contralateral to the ear receiving acoustic stimulation) at the appropriate location to gain access to the left LA, Str, HC, and Cg. The dura of the brain was removed and an electrode was inserted into the brain and advanced into the desired brain region using stereotaxic coordinates [72].

Broadband noise and tone bursts (50 ms duration, 1 ms rise/fall time, cosine^2^-gated) were generated (TDT RX6-2, ~100 kHz sampling rate) and presented at a rate of 2/s through a loudspeaker (FT28D, Fostex) located 10 cm in front of the right ear. Stimuli were calibrated using the electrical output from a sound level meter (Larson Davis model, 1/4 inch microphone, model 2520) which was delivered to a custom sound calibration program in the computer. Responses to the noise bursts were obtained at 11 intensities (0–100 dB SPL, 10-dB steps, 100 repetitions per intensity, pseudorandom presentation). Responses to tone bursts were collected at 10 frequencies (1.0, 1.5, 2.3, 3.5, 5.3, 8.0, 12.1, 18.3, 27.7, and 42.0 kHz) at 6 intensity levels (0–100 dB SPL, 20-dB steps, 50 repetitions per frequency-intensity combination, pseudorandom presentation order).

Local field potentials (LFPs) and spike discharges were sampled simultaneously from the same electrode with a resolution of 40.96 *μ*s using a RA16PA preamplifier and RX5 base station (Tucker-Davis Technologies System-3, Alachua, FL) using custom-written data acquisition and analysis software (MATLAB R2007b, MathWorks) as previously described [13, 56]. Following digital bandpass filtering (2–300 Hz), LFP signals were down-sampled online to 610 Hz. Averaged evoked LFPs were computed from the down-sampled data over a 500 ms time window following stimulus onset. Spike detection was performed online using a manually set voltage threshold (spike signal filtered 300–3500 Hz). Peristimulus time histograms (PSTH) were constructed offline using custom software with a time window up to 500 ms and bin widths of 1–10 ms. The root mean square (RMS) of LFP was measured and mean discharge rate of neuronal activity was obtained in a time window of 0–100 ms.

### 2.9. Anatomical Confirmation of Electrode Position

**Figure 1:**
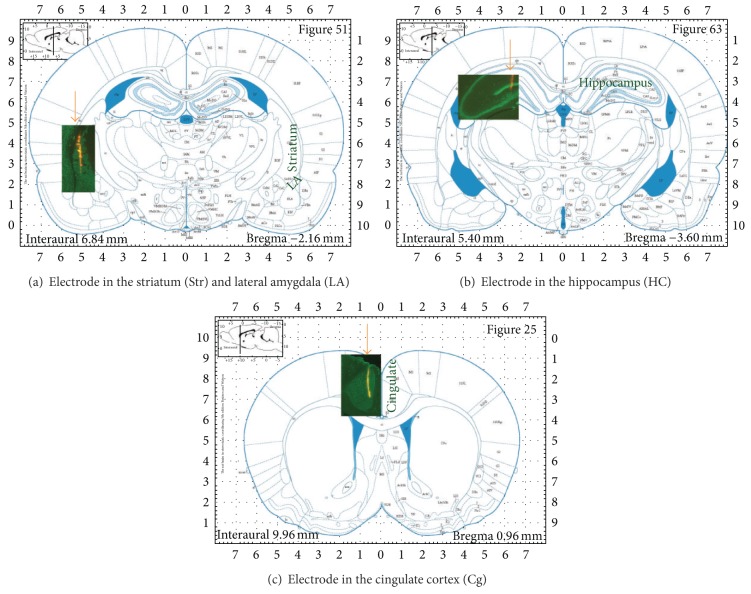
The recording electrodes in the brain. The drawings of the brain coronal section are from the rat brain atlas [72] and the inserts are photomicrographs of the brain showing DiI labeling of the recording electrodes (pointed by the arrows) in the Str and LA (a), the HC (b), and the Cg (c).

In addition to stereotaxic coordinates, the electrode position in the brain was verified in at least 2 animals per recording site by painting a fluorescent dye (DiI, Cat no. 42364, Sigma-Aldrich) on the surface of the electrode prior to penetration. After completing the recordings, the brain was removed, placed in 10% buffered formalin for 5–7 days, and immersed in 30% sucrose solution for two days. The brain was cryosectioned (50 *μ*m) in the coronal plane. After blocking in normal horse serum, slices were incubated in a primary mouse antineuronal nuclei (NeuN) monoclonal antibody (1 : 1000, Chemicon, MAB377), washed three times with phosphate buffered saline (PBS), and incubated with a donkey anti-mouse secondary antibody conjugated to Alexa Fluor 488 (1 : 1000; Invitrogen, A21202). Sections were washed with PBS and mounted on Fisher Superfrost polarized slides and coverslipped with Prolong Antifade mounting medium (Invitrogen). Sections were visualized and photographed with a Zeiss Axio Imager Z1 Microscope equipped with a digital camera, and images were processed with Zeiss AxioVision software.  Figure 1 presents the electrode penetration locations (pointed by arrows) in the Str-amygdala (a), HC (b), and Cg (c).

### 2.10. Statistical Analysis

One- and two-way ANOVAs (GraphPad ver. 5, Prism) and* t*-tests were used to evaluate the significance of the results.

## 3. Results

### 3.1. Behavioral Changes after SS Injection

#### 3.1.1. Hearing Loss

**Figure 2:**
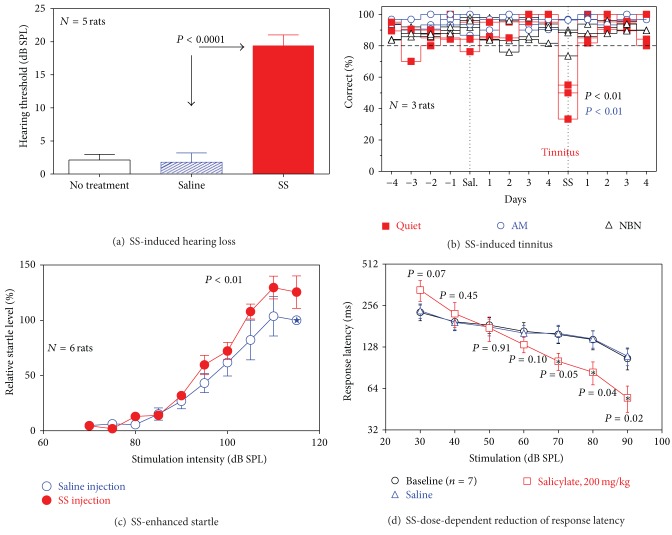
The effects of sodium salicylate (SS) injection on auditory perception. (a) Mean hearing thresholds for broadband noise bursts (*n* = 5). Baseline (black open bar), saline (blue shadowed bar), and salicylate (red filled bar) conditions are shown with standard error (SE) bars. Thresholds significantly increased by about 17 dB following salicylate administration; (b) salicylate-induced tinnitus. Rats (*n* = 3) were trained to respond to 3 stimuli. Quiet (red filled squares) and amplitude modulated (AM) (blue open circles) stimuli were paired with the right feeder while a narrowband noise (NBN) (black open triangles) was paired with the left feeder. Injection of saline (sal) showed no significant difference in responding during Quiet trials compared with baseline (no injection). However, an injection of 200 mg/kg SS showed a significant difference in response only during Quiet trials. This switch in response suggests that the rats perceived a steady state sound in the absence of an acoustic source. (c) Mean percentage (±SE) of startle amplitude relative to saline control startle amplitudes at 115 dB (marked with the star); note significantly increased startle amplitudes after salicylate injection, (d) Mean reaction time measures for broadband noise bursts (*n* = 7). Baseline (black circles), saline (blue triangles), and salicylate (red squares) are shown with standard error (SE) bars. Reaction times for 70, 80, and 90 dB SPL noise bursts decreased significantly with salicylate, suggesting an increased sensitivity to loud sounds.

To determine the magnitude of hearing loss resulting from SS treatment, five rats were trained to detect broadband noise bursts for a food reward. Normal untreated rats and saline treated rats could detect broadband noise bursts at ~2 dB SPL. However, after SS-injection, mean threshold was shifted to 19.4 dB SPL (Figure 2(a)). A one-way repeated measures ANOVA showed a significant difference between the treatments (*P* < 0.0001) and a Newman-Keuls Multiple Comparison Test showed that thresholds during SS treatment were significantly higher (~17.4 dB) than saline treatment (*P* < 0.0001).

#### 3.1.2. Tinnitus

To confirm that tinnitus-like behavior was present after SS treatment, three rats were trained on our two-alternative forced choice paradigm to activate a left feeder in the presence of a steady NBN and to activate the right feeder in the presence of an AM noise or no sound (Quiet).  Figure 2(b) presents the percentage of correct responses for each animal on AM, Quiet, and NBN trials 4 days prior to saline treatment (−4 to −1 days), ~2-3 h after saline treatment, days 1–4 after saline treatment, ~2-3 h after SS treatment, and days 1–4 after SS treatment. Prior to treatment (baseline control), the mean percentages of correct responses for NBN, AM, and Quiet trials were typically greater than 80% and never less than 70% correct for all three rats over the three conditions and four days; these results indicate that behavior was under stimulus control. Mean percentages of correct responses on NBN and AM trials were typically greater than 75% on the day of saline and greater than 80% on days 1–4 following saline treatment; these results indicate that animal behavior remained under stimulus control on the day of and the 4 days after saline treatment. Importantly, animals did not show a shift in responding during Quiet trials when treated with saline. However, on the day of SS treatment all three rats showed a dramatic change in their behavior on Quiet trials by shifting their response from the feeder associated with Quiet and AM to the feeder associated with a steady NBN, behavior consistent with hearing a steady phantom sound on Quiet trials rather than no sound. On days 1–4 following SS treatment, the percentages of correct responses reverted to 80% or more on Quiet trials behavior consistent with the absence of tinnitus. A repeated measures ANOVA showed significant differences on the Quiet trials between the saline treatment and SS treatment (*P* = 0.0066); a Newman-Keuls Multiple Comparison Test showed significant differences between Quiet and AM trials (*P* < 0.01) (i.e., fewer responses to the previously reinforced feeder on Quiet trials than during SS compared to saline) and between Quiet and NBN tests (*P* < 0.01) (i.e., a greater number of responses on Quiet trials to the NBN feeder during SS treatment compared to saline). Taken together, these results indicate that the rats can correctly discriminate AM and steady NBN during SS treatment; however, on roughly 45–65% of the Quiet trials the three rats mistakenly selected the feeder associated with a steady NBN suggesting that the rats are experiencing a phantom sound similar to the NBN stimulus.

#### 3.1.3. Startle Response

To determine if SS treatment would alter the rat's suprathreshold response to sound, we measured startle reflex response amplitudes in six rats to broadband noise bursts (20 ms) presented at intensities from 70 to 115 dB SPL. For ease of comparison, startle reflex amplitudes in each animal were normalized to the startle reflex response measured at 115 dB SPL (star) during the saline-control condition.  Figure 2(c) presents mean startle responses of the animals after saline control treatment (blue open circles) and after SS-injection (250 mg/kg, red filled circles). A two-way ANOVA (matching by rows) showed that the startle amplitudes in the SS-treated group were significantly larger than in the saline-treated group (*P* = 0.009).

#### 3.1.4. Loudness Perception

Previous researchers have used reaction time to estimate loudness perception in humans [7], monkeys [73], canaries [74], and cats [75]. Therefore, to confirm that hyperacusis-like behavior was present in our rats following an injection of SS, we measured reaction times to suprathreshold broadband noise bursts.  Figure 2(d) shows mean reaction times for 7 rats for baseline (no injection), saline (control), and SS (200 mg/kg, i.p.) treatments. There were no significant differences between baseline and saline reaction times; but after SS treatment, the rats exhibited significantly faster reaction times to 70 (*P* = 0.05), 80 (*P* = 0.04), and 90 dB SPL (*P* = 0.02) noise bursts than after saline treatment. As in humans with hyperacusis, animals with hyperacusis-like behavior showed shorter than normal reaction times for suprathreshold stimuli, presumably because sound stimuli are perceived as being louder than in normal-hearing animals [74]. In other words, rats given an injection of SS became more sensitive to loud sounds.

### 3.2. Neurophysiological Changes in the Brain after SS Injection

#### 3.2.1. Striatum

**Figure 3:**
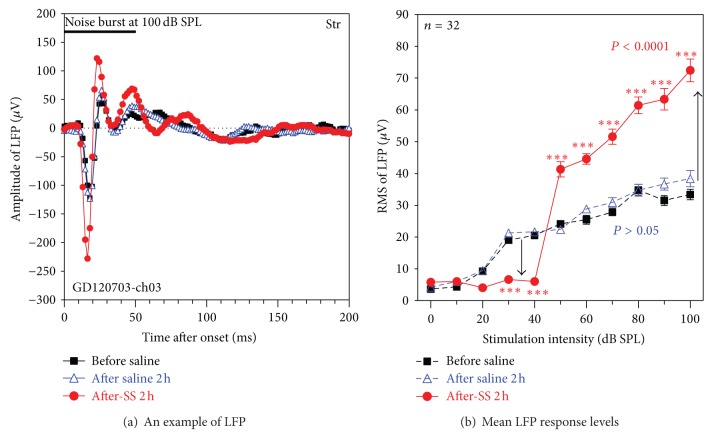
The effects of SS-injection on sound-evoked local field potential (LFP) elicited from electrodes in the Str. (a) An example of LFP at 100 dB SPL recoded before treatment (black filled squares), after saline injection (blue open triangles), and after SS injection (red filled triangles), showing an enhanced response following SS-injection. (b) Mean RMSs of LFP (*n* = 32) in a time window of 100 ms as a function of stimulation level, showing progressive increase of LFP amplitude at high stimulation levels but a reduction at low stimulation levels. Acoustic stimulation: 50 ms noise burst; treatments: saline (5 mL/kg, i.p.) and SS (250 mg/kg, i.p.); the vertical bars are standard errors (SEs) and the ∗∗∗ means *P* < 0.001; the arrows indicate increase and decrease of LFP amplitude.

Previous studies indicate that some cells in the Str respond to sounds [76] that electrical stimulation of the Str can trigger phantom auditory percepts [61] and that SS induces c-fos expression in some cells in the Str [63]. To determine if and how SS altered its electrophysiological properties, we recorded from the Str before and after administering SS (250 mg/kg i.p.). Noise bursts (50 ms, 100 dB SPL) evoked a robust LFP in the Str (Figure 3(a)) with a large negative peak around 18 ms followed by positive peak near 23 ms. There was little or no change in the amplitude or waveform of the LFP 2 h after saline treatment. However, the negative and positive peaks of the Str LFP increased substantially 2 h after SS. To compare the LFP responses before and after the treatments, the RMS of the LFP was measured from 0 to 100 ms.  Figure 3(b) presents the mean RMS of LFP of 32 recordings as a function of intensity; mean data are shown before saline, 2 h after saline, and 2 h after SS. Saline treatment had no effect on LFP amplitudes. In contrast, the LFP input/output function 2 h after SS was shifted roughly (20 dB) to the right, indicative of a threshold shift (hearing loss) and consistent with the behavioral threshold shift (Figure 2(a)). In addition, LFP amplitudes at 50 dB SPL or higher were roughly twice as large as presaline values. A two-way ANOVA showed that SS induced significant changes in LFP amplitude (*P* < 0.0001). Bonferroni posttests revealed a significant decrease of LFP at 30–40 dB 2 h after SS treatment and a significant increase from 50 to 100 dB SPL (*P* < 0.001).

**Figure 4:**
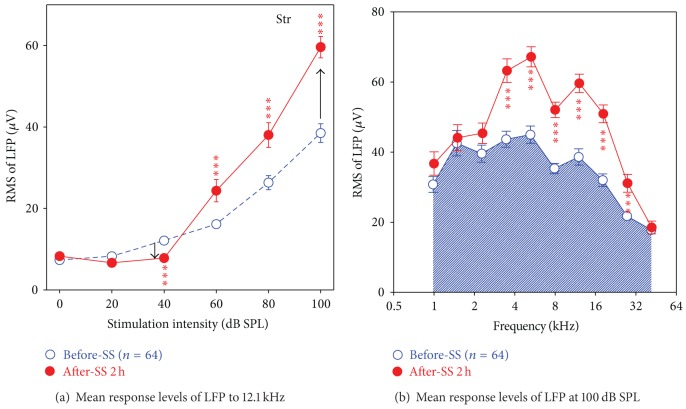
The effects of SS-injection on the tone-evoked LFP in the Str. (a) Mean RMSs of LFP (*n* = 64) to 12.1 kHz in a time window of 100 ms as a function of stimulation level, showing increase of LFP amplitude at high stimulation levels but a reduction at low stimulation levels. (b) Mean RMSs of LFP (*n* = 64) at 100 dB SPL as a function of stimulation frequency, showing significant enhancement at midfrequencies (3.5–18.3 kHz). Acoustic stimulation: 50 ms tone bursts at different frequencies; treatment: SS (250 mg/kg, i.p.); the vertical bars are SEs and the ∗∗∗ means *P* < 0.001; the arrows indicate increase and decrease of LFP amplitude.

Figure 4 demonstrates the effects of SS injection (250 mg/kg) on tone burst-evoked LFP in the Str of five rats (64 recordings from different Str locations). LFP response to midfrequency tones (Figure 4(a), 12.1 kHz as an example) increased at high stimulus levels (>50 dB SPL) and decreased at stimulus levels <50 dB SPL (Figure 4(a)) consistent with the noise-burst LFP. To determine if the changes in LFP amplitude were frequency dependent, LFP amplitudes were measured at 100 dB SPL before and 2 h after SS is plotted as a function of frequency (Figure 4(b)). Pretreatment LFPs were largest at low frequencies (1.5–8 kHz) and decreased at high frequencies (see blue open circles). However, 2 h after the SS injection, midfrequency region (3.5–18.3 kHz) LFPs were much larger than normal, whereas low-frequency and high-frequency LFPs showed much smaller increases (two-way ANOVA, Bonferroni posttests, *P* < 0.001). Similar results were observed at other stimulation levels (data not shown). These results are consistent with our previous observation of SS-induced hyperactivity at the midfrequencies in the inferior colliculus (IC) [14].

**Figure 5:**
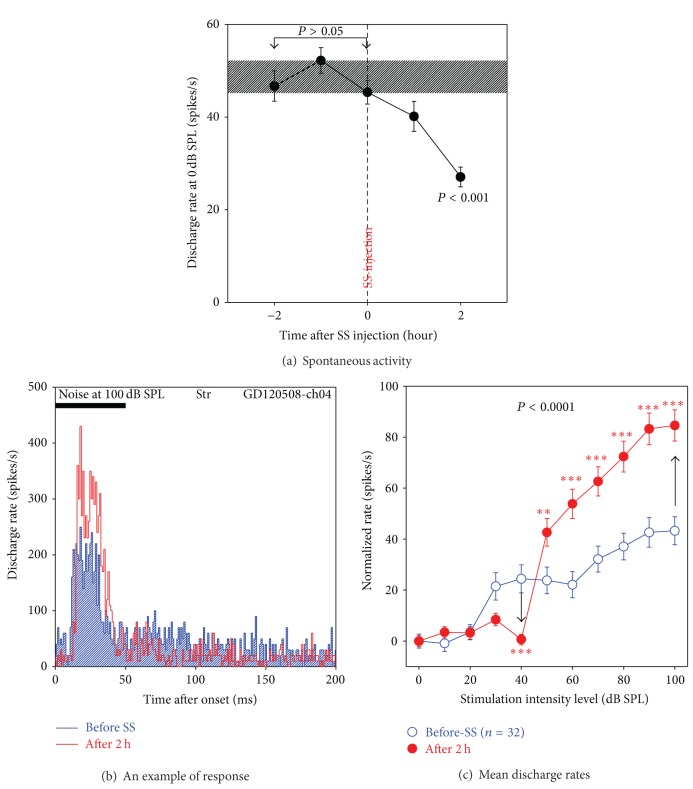
The SS effects on unit activity of neurons in the Str. (a) Mean spontaneous discharge rates (*n* = 32) as a function of time showing significant decrease after SS injection (*P* < 0.001). (b) An example of peristimulus time histograms (PSTH) obtained before SS (blue) and after SS (red), showing SS-induced increase of discharge rate. (c) Averaged discharge rates of neurons in the Str (*n* = 32) in a time window of 100 ms, showing a similar effect of SS injection as the LFP recorded in the nucleus. The discharge rates of each neuron were normalized to that at 0 dB SPL. Acoustic stimulation: 50 ms noise burst; treatment: SS (250 mg/kg, i.p.); the vertical bars are SEs; the arrows indicate increase and decrease of unit activity; ***P < 0.001; **P < 0.01.

Figure 5(a) presents the mean spontaneous discharge rates of 32 multiunit clusters in the Str measured before (−2 h to 0 h) and after SS treatment (1 h, 2 h). Spontaneous rates were stable before SS treatment (−2 h to 0 h; one-way ANOVA, Newman-Keuls Multiple Comparison Tests, *P* > 0.05); the mean rate (±SEM) during the pretreatment period is shown by the dashed horizontal rectangle. Spontaneous rates began to decline 1 h after SS and were significantly below pretreatment firing rates 2 h after SS (*P* < 0.001).

Figure 5(b) shows a representative PSTH of a multiunit cluster in the Str in response to a noise burst (50 ms duration, 100 dB SPL). The firing rate was enhanced after SS injection (red PSTH above blue PSTH) resulting in a sharper onset peak. Because SS reduced spontaneous activity, the transient nature of the onset response was further accentuated by the reduced spontaneous rate prior to the onset response (red line below blue line 0–10 ms).  Figure 5(c) presents the mean discharge rates of 32 multiunit clusters as a function of intensity. Since spontaneous activity decreased after the SS treatment, the sound-evoked discharge rate was normalized by subtracting the mean firing rate at 0 dB SPL from the mean firing rate at higher intensities. The normalized discharge rates represent the sound-driven responses. Similar to the LFP, the sound-evoked discharge rates were enhanced at intensities ≥50 dB SPL but reduced at levels <50 dB SPL leading to a threshold shift of ~20 dB. A two-way ANOVA showed a significant change pre- and post-SS injection (*P* < 0.0001). The sound-evoked firing rate 2 h after SS was significantly below pretreatment values at 40 dB SPL, whereas firing rates 2 h after SS were significantly greater than normal from 50 to 100 dB SPL (Figure 5(c); Bonferroni posttests, ***P < 0.001, **P < 0.01).

**Figure 6:**
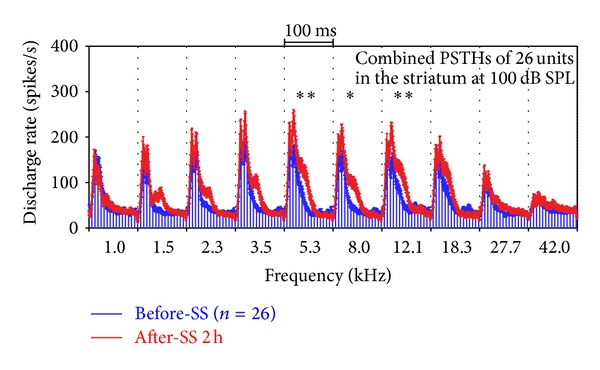
Averaged PSTHs of 26 units recorded in the Str showing significant effect (enhancement) in the midfrequency region (5.3, 8.0, and 12.1 kHz). The PSTHs were obtained before (blue) and after SS injection (red). Stimulation: 50 ms tone bursts at 100 dB SPL and at different frequencies; treatment: SS (250 mg/kg, i.p.). The vertical bars are SEs. **P < 0.01; *P < 0.05.

Similar to the tone-evoked LFP (Figure 4(b)), tone-evoked firing rates of multiunit clusters were affected in a frequency dependent manner after the SS-injection. To identify the SS-induced changes in the population of units from which we recorded, we computed the mean PSTH at each frequency-intensity combination from all 26 multiunit clusters as described previously [56].  Figure 6 presents the mean PSTHs (100 ms duration, 5 ms bin width) of 26 multiunit clusters obtained in the Str in response to 50 ms tone bursts presented at 100 dB SPL. Control responses are shown in blue; responses obtained 2 h after SS (250 mg/kg) are shown in red. The mean discharge rates of the PSTH were enhanced 2 h after SS. The firing rate increases, which was most pronounced between 1.5 and 18.3 kHz, resulting in a larger onset peak and a prolongation of the PSTH. To quantify the changes, mean discharge rates were calculated from 0 to 100 ms of each mean PSTH; SS treatment produced a significant increase in firing rate at 5.3, 8.0, and 12.1 kHz (one-way ANOVA, Newman-Keuls multiple comparison test). Altogether, SS increased sound-evoked activity at high sound levels predominantly at the midfrequencies, increased threshold, but decreased spontaneous activity and sound-evoked activity at low sound levels.

#### 3.2.2. Lateral Amygdala

**Figure 7:**
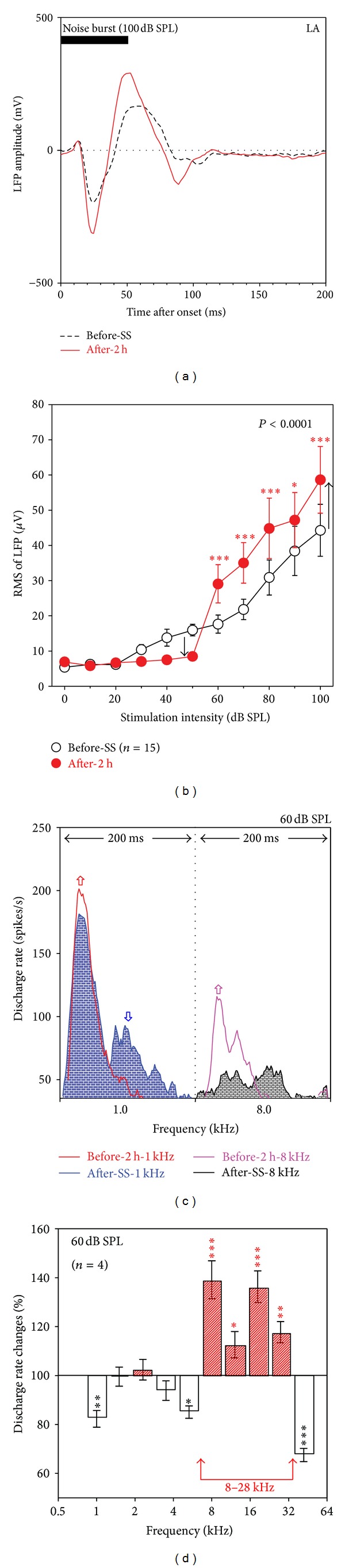
The effects of SS injection on auditory responses of the LA. (a) An example of LFP to noise burst at 100 dB SPL (black) showing an increase after SS injection (red). (b) Mean RMSs of LFP (*n* = 15) in a time window of 100 ms as a function of stimulation level, showing enhancement at high stimulation levels ≥60 dB SPL but reduction at low-stimulation levels <60 dB SPL. (c) Example of PSTHs in response to tones at 1.0 kHz (left) and 8.0 kHz (right) before and after SS injection showing greater increase after SS injection at the high-frequency; (d) SS-induced changes (%) of mean discharge rate in a time window of 100 ms showing SS-induced increase in the frequency range of 8–27.7 kHz. Stimulation: 50 ms noise or tone bursts; treatment: SS (250 mg/kg, i.p.). The vertical bars are SEs. *P < 0.05, **P < 0.01, and ***P < 0.001.

The amygdala, which assigns emotional significance to past experiences, has been implicated in tinnitus and hyperacusis [59, 63, 77], but its precise role in SS-induced tinnitus is poorly understood. Therefore we recorded from the LA to determine how SS would influence its electrophysiological properties. Noise-burst-evoked LFP from the LA had a longer latency, longer duration, and broader peaks than those from the Str (Figure 7(a)). The LFP from the LA evoked by a 100 dB SPL noise burst (black) consisted of a negative peak at ~25 ms and a positive peak at ~55 ms. The sound-evoked LFP increased substantially after SS injection (250 mg/kg, red line) and the peaks became narrower. The mean LFP amplitude-intensity function from the LA evoked by noise bursts is shown in Figure 7(b) before and 2 h after SS. SS treatment resulted in a slight-to moderate reduction in response amplitude at low intensities (<60 dB SPL), a threshold shift of approximately 20 dB, and a significant increase in response amplitude at high intensities (≥60 dB) (two-way ANOVA, Bonferroni post hoc tests significant at ≥60 dB SPL).

Figure 7(c) shows mean PSTHs of 4 multiunit clusters recorded in the LA in response to 1.0 kHz and 8.0 kHz tone bursts presented at 60 dB SPL. The 1.0 kHz tone induced a robust response (blue) with a large, narrow, short-latency peak followed by a smaller, long latency peak. SS induced striking changes in the temporal profile of the 1 kHz PSTH; the early part of the response was slightly enhanced while the late part of the response was completely suppressed. The mean PSTH to the 8.0 kHz, 60 dB tone burst was broad and lacked a sharp onset response (Figure 7(c), black); however, 2 h after SS injection, the same tone burst evoked a more robust response with a completely different temporal PSTH profile, one that consisted of a large, sharp onset peak followed by the loss of the delayed response (Figure 7(c), purple). To quantify the frequency effect of SS, mean discharge rates during SS treatment were normalized to the pretreatment firing rate and expressed as percentage of pretreatment rate (Figure 7(d)). At low frequencies (1.0–5.3 kHz) and a high frequency (42.0 kHz), the mean discharge rates either remained near pretreatment control levels (~100%, 1.5–3.5 kHz) or were significantly lower than the controls (<100%, 1.0, 5.3, and 42.0 kHz). In contrast, mean discharge rates at the midfrequencies (8.0–27.7 kHz) increased significantly (>100%).

The mean spontaneous rates of eight multiunit clusters increased slightly from 34.4 ± 21.1 spikes/s (mean ± SD) before treatment to 42.5 ± 20.4 spikes/s 1 h after SS; the increase did not reach statistical significance (*P* = 0.12,* t*-test).

#### 3.2.3. Hippocampus

**Figure 8:**
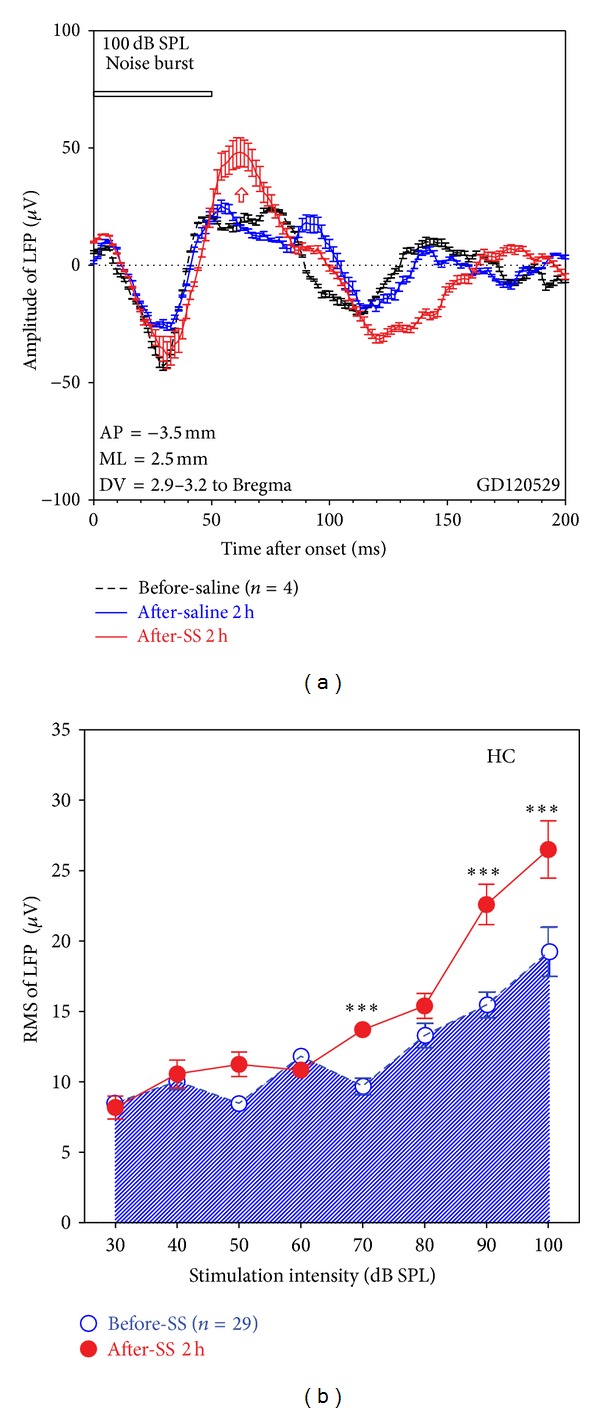
The effect of SS injection on noise-burst-evoked LFP elicited from electrodes in the hippocampus. (a) Averaged LFP (*n* = 4 recordings in one rat) at 100 dB SPL recoded before treatment (black), after saline injection (blue), and after SS injection (red), showing a slight increase after SS injection. (b) Mean RMSs of LFP (*n* = 29) in a time window of 100 ms as a function of stimulation level, showing enhancement at high stimulation levels. Stimulation: 50 ms noise burst; treatment: SS (250 mg/kg, i.p.). The vertical bars are SEs and the ∗∗∗ means *P* < 0.001.

The HC, important in memory formation, has been implicated in tinnitus [59, 78, 79], but its functional contributions to SS-induced tinnitus are unclear. To evaluate its contributions, we recorded from the HC in six rats before and after SS treatment. Broadband noise bursts induced a clear LFP in the HC but seldom evoked strong neuronal discharges. Responses to tone bursts were also weak; therefore, we focused our analysis on noise-burst-evoked LFP.  Figure 8(a) presents averaged LFP from 4 recordings obtained from electrodes in the dorsal HC of one animal in response to noise bursts. The LFP evoked by 100 dB noise bursts consisted of a broad negative peak around 30 ms followed by a much broader positive peak beginning around 50 ms (Figure 8(a), black). Saline treatment had little or no effect on the amplitude or profile of the LFP (Figure 8(a), blue). However, the amplitude of the LFP increased and the positive peak became narrower 2 h after SS treatment.  Figure 8(b) shows the RMS amplitude (100 ms window) of the LFP (*n* = 29) from the HC as a function of noise-burst intensity. Pretreatment LFP amplitudes increased slowly up to 70 dB SPL and then increased more rapidly at higher levels reaching a maximum of around 19 *μ*V at 100 dB SPL, much smaller than the LFP in the Str and LA. LFP amplitude increased significantly at 70, 90, and 100 dB SPL 2 h after SS treatment (two-way ANOVA, intensity a repeated measure, *P* < 0.0001; Bonferroni post hoc tests); the amplitude increase in the HC, about 35%, was less than in the Str and LA.

#### 3.2.4. Cingulate Cortex

**Figure 9:**
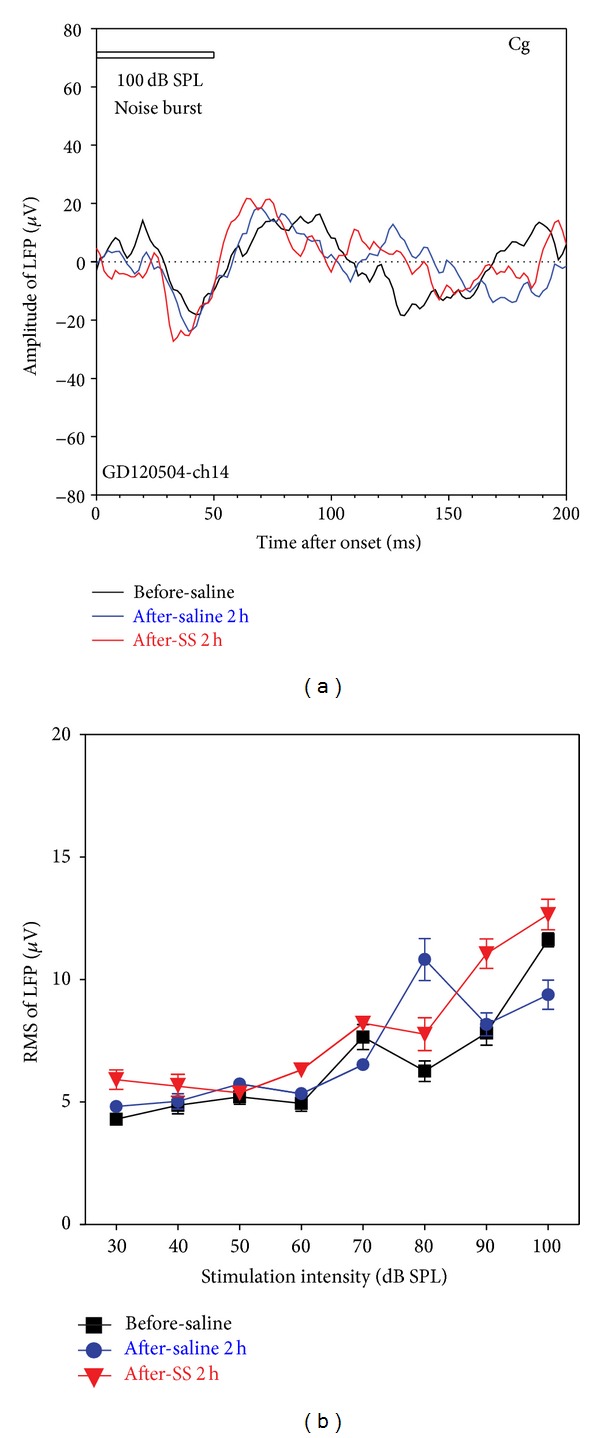
The effect of SS on noise-burst-evoked LFP elicited from electrodes in the cingulate cortex. (a) An example of LFP at 100 dB SPL recoded before treatment (black), after saline (blue), and after SS (red), showing no change during treatment. (b) Mean RMSs of LFP (*n* = 16) in a time window of 100 ms as a function of stimulation level, showing no significant change of the mean LFP. Stimulation: 50 ms noise burst; treatment: SS (250 mg/kg, i.p.). The vertical bars are SEs.

The Cg has been implicated in tinnitus distress and showed strong c-fos immunolabeling following salicylate treatment [63, 80, 81]. We recorded the LFP from the Cg to identify possible electrophysiological changes induced by SS. Noise-burst LFPs from the Cg were substantially smaller and broader than those from the HC, LA, and Str and few Cg multiunit clusters responded to tones or noise. Noise-burst-evoked LFPs were measured from the Cg of four rats. LFP waveforms varied with electrode depth. Upon penetrating area-1 of the cingulate Cg1 [72], an LFP was encountered with an initial positive peak (~35 ms latency; data not shown). With increasing electrode depth and entry into cingulate area-2 (Cg2), the LFP reversed polarity (Figure 9(a)) and increased amplitude. The LFP from Cg2 began with a negative peak (~35 ms latency) followed by an extremely broad positive peak (~70 ms latency). LFPs were measured from Cg2 region prior to treatment, 2h post-saline treatment, and 2h post-SS treatment (250 mg/kg). LFP amplitudes and waveforms remained largely unchanged after the saline (blue) and SS treatments (red, Figure 9(a)).  Figure 9(b) presents mean (RMS, 100 ms window, *n* = 16) noise burst versus intensity functions measured in the Cg before, 2 h after saline, and 2 h after SS (250 mg/kg) treatments. In contrast to the large amplitude increases observed in other areas (St, LA, and HC), LFP amplitude-intensity functions in the Cg were largely unaffected by SS treatments.

## 4. Discussion

### 4.1. Behavioral Features

SS has long been known to induce sensorineural hearing loss by affecting the electromotile response of cochlear outer hair cells and neural activity in the cochlea [2]. The magnitude of the hearing loss is related to SS dose and serum salicylate levels [82]. The 200 mg/kg SS dose increased noise-burst behavioral thresholds ~17.4 dB (Figure 2(a)). Our noise-burst threshold shifts are slightly greater (~7.5 dB) than those reported previously with the same dose of SS, but with a different behavioral method and low-to-mid frequency tone bursts instead of noise bursts [83]. Our noise-burst threshold shifts, however, were similar to the noise-burst LFP threshold shifts observed in the Str and LA.

Despite the threshold elevation and reduced neural output at low stimulus levels (Figures 3, 4, 5, and 7), SS enhanced the amplitude of acoustic startle reflex at high stimulus levels (Figure 2(c)), consistent with previous results [20] and reduced animals' reaction times to loud sounds (Figure 2(d)). The enhanced motor response to suprathreshold sounds could conceivably be related to hyperacusis, a perceptual phenomenon whereby high intensity sounds become intolerably loud, a condition that frequently accompanies tinnitus [84, 85]. However, this hyperactive motoric response may not be the perceptual equivalent of hyperacusis until further confirmatory data are obtained from human listeners with hyperacusis. Alternatively, the enhancement of suprathreshold startle reflex amplitudes could be related to the increased suprathreshold excitability seen within the central auditory pathway, as we have previously reported [8, 20, 86]. Greater neural activity within the LA, known to modulate the startle reflex [87, 88], and neural activity in the Str, which influences motor movements and vocalizations could enhance the startle reflex [51, 89–91].

Previous studies indicate that the minimum dose of SS needed to induce tinnitus in rats is 150–200 mg/kg [3, 4, 92]. In agreement with these earlier studies employing different techniques, we observed robust behavioral evidence of tinnitus on Quiet trials 2 h following the administration of 200 mg/kg SS; in contrast saline had no effect on Quiet performance. One day later, after SS washout, behavior on Quiet trials reverted to normal. Importantly, the performance of the rats to the steady NBN and the AM signal were unaffected by SS treatment indicating that the behavior remained under stimulus control. Taken together, the behavioral results confirm that our salicylate treatment induced mild, reversible hearing loss, tinnitus, increased sensitivity to suprathreshold sounds, and enhanced acoustic startle reflex motor activity to high intensity sounds.

### 4.2. SS and Nonauditory Structures

The ototoxic effects of SS on the cochlea have been well documented [9, 13, 20]; however, its effects on the central nervous system are only beginning to be explored, despite the fact that SS readily crosses the blood-brain barrier [93, 94]. The past two decades have seen a rapid increase in our understanding of how SS affects the function of neurons in the central auditory pathway, but comparatively little is known about the effects of SS on structures outside the classical auditory pathway. Insights likely affected brain structures can be gleaned from earlier c-fos immunolabeling studies [63, 95, 96]. Since SS increased c-fos labeling in the Str, LA, and Cg we investigated the electrophysiological changes in these areas along with the hippocampus where rather modest c-fos labeling occurred.

#### 4.2.1. Brain Gain

Acoustic stimulation induced robust neural response in the LA and Str consistent with earlier reports [23, 76, 97]. SS produced a number of well-defined changes in the LA and Str. LFP thresholds increased approximately 20 dB following SS treatment similar to behavior thresholds (Figures 2(a), 3(b), and 7(b)). The threshold elevation in the LA and Str is most likely due to a cochlear hearing loss which reduces the neural output of the cochlea [2, 13, 20]. Despite a reduced neural output from the cochlea after SS treatment [8], suprathreshold responses from the LA and Str were greatly enhanced (Figures 3 and 7) [56]. These results suggest that the neural output from the cochlea is amplified as it transits up the central nervous system. Previous reports indicate that LFPs in the inferior colliculus are nearly normal after SS treatment; this implies that some amplification is already occurring between the auditory nerve midbrain. Broadly speaking, neural amplification could result from increased excitation or decreased inhibition.* In vitro*, SS reduces *γ*-aminobutyric acid (GABA) mediated inhibitory currents in auditory cortex, hippocampus, inferior colliculus, and spinal neurons [10–12, 98] while acute SS treatment* in vivo* decreased GABA expression and increased glutamate expression in the inferior colliculus [99]. Since LFPs from the LA, Str, and HC, as well as auditory cortex and medial geniculate, become larger than normal after SS [8, 20], additional amplification, likely due to a reduction of GABA-mediated inhibition, must occur above the midbrain [20, 70]. Taken together, these results suggest that increased neural gain occurs at multiple sites within the central nervous system. In response to a reduced cochlear output, the central auditory system becomes more responsive to a reduced input indicative of increased central gain or sensory rescaling due to peripheral hearing loss.

The hyperexcitability in the Str and LA was frequency dependent, similar to that previously reported in auditory cortex [13]. Interestingly, tone evoked hyperactivity was maximal at the midfrequencies (Figures 4(b), 6, and 7(d)) where the pitch of SS-induced tinnitus occurs [100]. Salicylate is known to induce tinnitus with a pitch between 10 and 20 kHz [69, 101]. Physiologically, the CF of many neurons in the auditory cortex (AC) and LA shifted into the tinnitus frequency region after SS treatment [13, 56]. In the current report, enhancement of suprathreshold responses of the LA also occurred in the frequency range of 8–28 kHz (Figure 7(d)) and that of the Str occurred in the frequency range of 3.5–28 kHz (Figure 4(b)). The midfrequency hyperactivity could result from two factors. One is a cochlear frequency-dependent loss in sensitivity that was smallest at the midfrequencies and relatively greater at low and high frequencies [13]. The second is a SS-induced CF-shift in AC and LA such that many high-CF and low-CF neurons undergo a CF-shift towards the midfrequencies [13, 56]. One consequence of this CF shift is that many more neurons respond to the midfrequency tones than normal would do so.

#### 4.2.2. Temporal Profiles

SS altered the temporal profile of LFP and PSTH from the Str and LA. In general, the onset component of the LFP was more robust, the latency shorter, and the width narrower after SS treatment (Figures 3(a) and 7(a)). PSTH onset responses were more pronounced in the Str (Figures 5 and 6) and LA (Figure 7). SS had the opposite effects on the duration of the PSTH response in the Str and LA. In the Str, SS prolonged the duration of the response and in some cases generated a secondary peak with a latency around 50 ms (Figure 6, 1.2–3.5 kHz). The latency of this secondary peak corresponds closely with the pronounced increase in the second positive peak of the LFP from the Str (Figure 3(a)). An LFP can be evoked in the Str by electrical stimulation of overlying cortex [102]. The electrically evoked LFP consisted primarily of a single onset peak; however, when the GABAa receptor antagonist, bicuculline, was infused into the striatum, the initial peak of the LFP became larger and a secondary peak appeared in the LFP. In addition, bicuculline increased the number of action potentials and the duration of the response merges effects similar to those induced by SS [10–12, 98]. This suggests that the amplitude enhancement and prolongation of the response in the Str are due to a loss of local GABA-mediated inhibition. Disruption of this circuit could impair auditory temporal processing. Indeed, high doses of aspirin, whose active ingredient is salicylate, lead to a slight impairment of temporal resolution [103].

SS increased the amplitude of the onset response in the LA, decreased the latency of the second peak of the LFP, and reduced the duration of the PSTH so that the response was more phasic than sustained. SS enhanced the onset response and shortened the duration of responses in the supragranular layer of the auditory cortex [86], changes attributed to reduced intracortical GABA-medicated inhibition [70, 104]. Electrical stimulation of the medial geniculate body, part of the auditory pathway, evoked a negative-positive LFP in the LA. Administration of baclofen, a GABAb agonist, significantly reduced the amplitude and increased the latency of the positive peak [105]. Since SS suppresses GABA-mediated inhibition [12], its effects on the LA, either directly or indirectly, would be expected to increase the amplitude and decrease the latency of the positive peak similar to what we observed (Figure 7).

#### 4.2.3. Spontaneous Activity and Tinnitus

Models of tinnitus often assume that the phantom sound arises from an increase in spontaneous activity localized to the region of hearing loss and tinnitus pitch [106]. While there is a good deal of data in the auditory brainstem and midbrain to support this hypothesis for cases of chronic tinnitus [107–110], the effects of SS on spontaneous rates have varied across studies, region of the brain, and drug dose employed [14, 18, 19, 21, 56, 69, 111–113]. In this study we found a slight increase of spontaneous activity in the LA but a decrease in the striatum, which was inconsistent with the sound-evoked response. Our SS data suggest that different mechanisms modulate spontaneous activity and sound-driven responses in the Str. Although c-fos functional relationship to neuron firing is not well understood, c-fos immunolabeling has nevertheless often been used as a marker of neural activity [114]. Immunolabeling studies have identified many regions of strong c-fos expression after SS treatment. SS induced strong c-fos labeling in the LA; therefore, we assumed that spontaneous activity might increase in LA after SS treatment [63]. However, we found that SS induced an insignificant increase of spontaneous activity among LA neurons that responded to sound stimulation. Strong c-fos labeling was also reported in the Str after SS treatment but surprisingly SS caused a significant decrease in spontaneous activity among Str neurons that responded to sound (Figure 5(a)). Thus, our results do not provide any support for the hypothesis that tinnitus is due to an increase in spontaneous activity in LA or Str. Moreover, the data suggest that the SS-induced change in c-fos expression is not a good predictor of spontaneous firing rate. However, this interpretation should be tempered by the fact that our assessment of spontaneous rate was obtained only from acoustically responsive neurons; it is conceivable that spontaneous rates may have increased among acoustically unresponsive neurons. Moreover, the effects of the anesthetics used in our study may have masked the effects of SS on spontaneous activity.

#### 4.2.4. Hyperactivity and Hyperacusis

Among the most robust and consistent electrophysiological change induced by SS treatment is the enhancement of suprathreshold sound-evoked responses at multiple sites in the central auditory pathway [8, 20]. SS also enhanced sound-evoked responses in the Str, LA, and HC, regions outside the classical auditory pathway. One common factor that may be responsible for these enhanced neural responses is the SS-induced reduction of GABA-mediated inhibition [11, 12, 20, 99]. The robust increase in suprathreshold neural activity in several higher auditory centers could conceivably cause sounds to be perceived as much louder than normal (hyperacusis); this assumes that the amplitude of sound-evoked LFP is closely correlated with the loudness.

The Str is known to modulate the startle reflex [34, 115] and the SS-induced enhancement of neural activity in this motor area could conceivably contribute to the enhanced startle amplitudes (Figure 2(b)). Electrical stimulation of the amygdala can enhance the startle reflex [116]. Thus, the SS-induced enhancement of LA responses could be another factor that potentiates the startle reflex amplitude after SS. The HC also modulates the startle reflex [117]. Thus, the SS-induced enhancement of HC activity provides another means of increasing the startle responses. However, SS failed to enhance responses in the Cg indicating that this structure is unlikely to be involved with the startle response. The lack of functional change in Cg is rather surprising given that SS significantly increased c-fos labeling in this region [63].

### 4.3. Anesthesia

In this study, we administered SS to ketamine anesthetized rats. In an earlier study, we demonstrated that ketamine, noncompetitive NMDA antagonist, accentuated the SS-induced enhancement of sound-evoked activity in the auditory cortex [20]. Ketamine also enhanced the cortically generated 40 Hz auditory steady-state response but not the more peripherally generated auditory brainstem response [1, 118]. In contrast, isoflurane anesthesia, which enhances GABAergic activity, suppressed the SS-induced enhancement of the LFP [20]. Taken together, these results suggest that the SS-induced enhancement of sound-evoked activity may be due to its combined effects glutamatergic and GABAergic synapses.

## 5. Conclusion

Rats treated with SS doses of 200 and 250 mg/kg showed behavioral evidence of hearing loss and tinnitus and responded in a hyperactive manner to loud sounds. These SS-induced behavioral changes were accompanied by suprathreshold hyperexcitability in the Str, a motor area, the LA, an emotional center, and HC, involved in memory and spatial navigation. SS shortened temporal response in the LA, whereas in the Str it prolonged the response and reduced spontaneous activity. The SS-induced hyperactivity observed in the LA, Str, and HC implicates plastic change in the nuclei and may contribute to the enhancement of the startle reflex and hyperacusis.

## References

[1] Bobbin RP, May JG, Lemoine RL (1979). Effects of pentobarbital and ketamine on brain stem auditory potentials. Latency and amplitude intensity functions after intraperitoneal administration. *Archives of Otolaryngology*.

[2] Cazals Y (2000). Auditory sensori-neural alterations induced by salicylate. *Progress in Neurobiology*.

[3] Jastreboff PJ, Brennan JF, Coleman JK, Sasaki CT (1988). Phantom auditory sensation in rats: an animal model for tinnitus. *Behavioral Neuroscience*.

[4] Lobarinas E, Sun W, Cushing R, Salvi R (2004). A novel behavioral paradigm for assessing tinnitus using schedule-induced polydipsia avoidance conditioning (SIP-AC). *Hearing Research*.

[5] Kizawa K, Kitahara T, Horii A (2010). Behavioral assessment and identification of a molecular marker in a salicylate-induced tinnitus in rats. *Neuroscience*.

[6] Turner JG, Parrish J (2008). Gap detection methods for assessing salicylate-induced tinnitus and hyperacusis in rats. *American Journal of Audiology*.

[7] Arieh Y, Marks LE (2003). Recalibrating the auditory system: a speed-accuracy analysis of intensity perception. *Journal of Experimental Psychology: Human Perception and Performance*.

[8] Chen GD, Stolzberg D, Lobarinas E, Sun W, Ding D, Salvi R (2013). Salicylate-induced cochlear impairments, cortical hyperactivity and re-tuning, and tinnitus. *Hearing Research*.

[9] Santos-Sacchi J, Song L, Zheng J, Nuttall AL (2006). Control of mammalian cochlear amplification by chloride anions. *The Journal of Neuroscience*.

[10] Gong N, Zhang M, Zhang X-B, Chen L, Sun G-C, Xu T-L (2008). The aspirin metabolite salicylate enhances neuronal excitation in rat hippocampal CA1 area through reducing GABAergic inhibition. *Neuropharmacology*.

[11] Wang H-T, Luo B, Zhou K-Q, Xu T-L, Chen L (2006). Sodium salicylate reduces inhibitory postsynaptic currents in neurons of rat auditory cortex. *Hearing Research*.

[12] Xu M, Gong N, Chen L, Xu T-L (2005). Sodium salicylate reduces gamma aminobutyric acid-induced current in rat spinal dorsal horn neurons. *NeuroReport*.

[13] Stolzberg D, Chen G-D, Allman BL, Salvi RJ (2011). Salicylate-induced peripheral auditory changes and tonotopic reorganization of auditory cortex. *Neuroscience*.

[14] Chen G-D, Jastreboff PJ (1995). Salicylate-induced abnormal activity in the inferior colliculus of rats. *Hearing Research*.

[15] Eggermont JJ, Kenmochi M (1998). Salicylate and quinine selectively increase spontaneous firing rates in secondary auditory cortex. *Hearing Research*.

[16] Kenmochi M, Eggermont JJ (1997). Salicylate and quinine affect the central nervous system. *Hearing Research*.

[17] Ochi K, Ohashi T, Kato I, Eggermont JJ (1997). Effects of salicylate and quinine on cat primary auditory cortex—spontaneous firing rate. *Journal of Otolaryngology of Japan*.

[18] Ochi K, Eggermont JJ (1996). Effects of salicylate on neural activity in cat primary auditory cortex. *Hearing Research*.

[19] Jastreboff PJ, Sasaki CT (1986). Salicylate-induced changes in spontaneous activity of single units in the inferior colliculus of the guinea pig. *Journal of the Acoustical Society of America*.

[20] Sun W, Lu J, Stolzberg D (2009). Salicylate increases the gain of the central auditory system. *Neuroscience*.

[21] Zhang X, Yang P, Cao Y, Qin L, Sato Y (2011). Salicylate induced neural changes in the primary auditory cortex of awake cats. *Neuroscience*.

[22] LeDoux JE, Ruggiero DA, Reis DJ (1985). Projections to the subcortical forebrain from anatomically defined regions of the medial geniculate body in the rat. *The Journal of Comparative Neurology*.

[23] LeDoux JE, Farb C, Ruggiero DA (1990). Topographic organization of neurons in the acoustic thalamus that project to the amygdala. *The Journal of Neuroscience*.

[24] LeDoux JE, Farb CR, Romanski LM (1991). Overlapping projections to the amygdala and striatum from auditory processing areas of the thalamus and cortex. *Neuroscience Letters*.

[25] Turner BH, Herkenham M (1991). Thalamoamygdaloid projections in the rat: a test of the amygdala’s role in sensory processing. *The Journal of Comparative Neurology*.

[26] Romanski LM, Clugnet M-C, Bordi F, LeDoux JE (1993). Somatosensory and auditory convergence in the lateral nucleus of the amygdala. *Behavioral Neuroscience*.

[27] LeDoux JE (2000). Emotion circuits in the brain. *Annual Review of Neuroscience*.

[28] Budinger E, Heil P, Hess A, Scheich H (2006). Multisensory processing via early cortical stages: connections of the primary auditory cortical field with other sensory systems. *Neuroscience*.

[29] Budinger E, Laszcz A, Lison H, Scheich H, Ohl FW (2008). Non-sensory cortical and subcortical connections of the primary auditory cortex in Mongolian gerbils: bottom-up and top-down processing of neuronal information via field AI. *Brain Research*.

[30] LeDoux J (2007). The amygdala. *Current Biology*.

[31] Damasio AR, Damasio H, Rizzo M (1982). Aphasia with nonhemorrhagic lesions in the basal ganglia and internal capsule. *Archives of Neurology*.

[32] Aram DM, Ekelman BL, Rose DF (1985). Verbal and cognitive sequelae following unilateral lesions acquired in early childhood. *Journal of Clinical Neuropsychology*.

[33] Klein D, Zatorre RJ, Milner B, Meyer E, Evans AC (1994). Left putaminal activation when speaking a second language: evidence from PET. *NeuroReport*.

[34] Kodsi MH, Swerdlow NR (1994). Quinolinic acid lesions of the ventral striatum reduce sensorimotor gating of acoustic startle in rats. *Brain Research*.

[35] Kodsi MH, Swerdlow NR (1995). Prepulse inhibition in the rat is regulated by ventral and caudodorsal striato-pallidal circuitry. *Behavioral Neuroscience*.

[36] Quirk GJ, Repa JC, LeDoux JE (1995). Fear conditioning enhances short-latency auditory responses of lateral amygdala neurons: parallel recordings in the freely behaving rat. *Neuron*.

[37] McKernan MG, Shinnick-Gallagher P (1997). Fear conditioning induces a lasting potentiation of synaptic currents in vitro. *Nature*.

[38] Quirk GJ, Armony JL, LeDoux JE (1997). Fear conditioning enhances different temporal components of tone-evoked spike trains in auditory cortex and lateral amygdala. *Neuron*.

[39] Boutros NN, Belger A (1999). Midlatency evoked potentials attenuation and augmentation reflect different aspects of sensory gating. *Biological Psychiatry*.

[40] Doupe AJ, Kuhl PK (1999). Birdsong and human speech: common themes and mechanisms. *Annual Review of Neuroscience*.

[41] Swerdlow NR, Braff DL, Geyer MA (1999). Cross-species studies of sensorimotor gating of the startle reflex. *Annals of the New York Academy of Sciences*.

[42] Shu SY, Wu YM, Bao XM (2002). A new area in the human brain associated with learning and memory: immunohistochemical and functional MRI analysis. *Molecular Psychiatry*.

[43] Tsvetkov E, Carlezon WA, Benes FM, Kandel ER, Bolshakov VY (2002). Fear conditioning occludes LTP-induced presynaptic enhancement of synaptic transmission in the cortical pathway to the lateral amygdala. *Neuron*.

[44] Krause M, Hoffmann WE, Hajós M (2003). Auditory sensory gating in hippocampus and reticular thalamic neurons in anesthetized rats. *Biological Psychiatry*.

[45] Cromwell HC, Anstrom K, Azarov A, Woodward DJ (2005). Auditory inhibitory gating in the amygdala: single-unit analysis in the behaving rat. *Brain Research*.

[46] Cromwell HC, Klein A, Mears RP (2007). Single unit and population responses during inhibitory gating of striatal activity in freely moving rats. *Neuroscience*.

[47] Cromwell HC, Woodward DJ (2007). Inhibitory gating of single unit activity in amygdala: effects of ketamine, haloperidol, or nicotine. *Biological Psychiatry*.

[48] Howland JG, Hannesson DK, Barnes SJ, Phillips AG (2007). Kindling of basolateral amygdala but not ventral hippocampus or perirhinal cortex disrupts sensorimotor gating in rats. *Behavioural Brain Research*.

[49] LeDoux J (2003). The emotional brain, fear, and the amygdala. *Cellular and Molecular Neurobiology*.

[50] LeDoux JE (1993). Emotional memory systems in the brain. *Behavioural Brain Research*.

[51] Baldan Ramsey LC, Xu M, Wood N, Pittenger C (2011). Lesions of the dorsomedial striatum disrupt prepulse inhibition. *Neuroscience*.

[52] Heutink J, Brouwer WH, de Jong BM, Bouma A (2011). Conscious and unconscious processing of fear after right amygdala damage: a single case ERP-study. *Neurocase*.

[53] An B, Hong I, Choi S (2012). Long-term neural correlates of reversible fear learning in the lateral amygdala. *The Journal of Neuroscience*.

[54] Hong I, Kim J, Song B (2012). Fear conditioning occludes late-phase long-term potentiation at thalamic input synapses onto the lateral amygdala in rat brain slices. *Neuroscience Letters*.

[55] Kazama AM, Heuer E, Davis M, Bachevalier J (2012). Effects of neonatal amygdala lesions on fear learning, conditioned inhibition, and extinction in adult macaques. *Behavioral Neuroscience*.

[56] Chen G-D, Manohar S, Salvi R (2012). Amygdala hyperactivity and tonotopic shift after salicylate exposure. *Brain Research*.

[57] Jastreboff PJ (2007). Tinnitus retraining therapy. *Progress in Brain Research*.

[58] Rauschecker JP, Leaver AM, Mühlau M (2010). Tuning out the noise: limbic-auditory interactions in tinnitus. *Neuron*.

[59] De Ridder D, Fransen H, Francois O, Sunaert S, Kovacs S, Van De Heyning P (2006). Amygdalohippocampal involvement in tinnitus and auditory memory. *Acta Oto-Laryngologica. Supplementum*.

[60] Cheung SW, Larson PS (2010). Tinnitus modulation by deep brain stimulation in locus of caudate neurons (area LC). *Neuroscience*.

[61] Larson PS, Cheung SW (2012). Deep brain stimulation in area LC controllably triggers auditory phantom percepts. *Neurosurgery*.

[62] Larson PS, Cheung SW (2013). A stroke of silence: tinnitus suppression following placement of a deep brain stimulation electrode with infarction in area LC. *Journal of Neurosurgery*.

[63] Wallhäusser-Franke E, Mahlke C, Oliva R, Braun S, Wenz G, Langner G (2003). Expression of c-fos in auditory and non-auditory brain regions of the gerbil after manipulations that induce tinnitus. *Experimental Brain Research*.

[64] Mahlke C, Wallhäusser-Franke E (2004). Evidence for tinnitus-related plasticity in the auditory and limbic system, demonstrated by arg3.1 and c-fos immunocytochemistry. *Hearing Research*.

[65] Stolzberg D, Hayes SH, Kashanian N, Radziwon K, Salvi RJ, Allman BL (2013). A novel behavioral assay for the assessment of acute tinnitus in rats optimized for simultaneous recording of oscillatory neural activity. *Journal of Neuroscience Methods*.

[66] Gellermann LW (1933). Chance orders of alternating stimuli in visual discrimination experiments. *Pedagogical Seminary and Journal of Genetic Psychology*.

[67] Heffner HE (2011). A two-choice sound localization procedure for detecting lateralized tinnitus in animals. *Behavior Research Methods*.

[68] Pilz PK, Schnitzler HU, Menne D (1987). Acoustic startle threshold of the albino rat (Rattus norvegicus). *Journal of Comparative Psychology*.

[69] Yang G, Lobarinas E, Zhang L (2007). Salicylate induced tinnitus: behavioral measures and neural activity in auditory cortex of awake rats. *Hearing Research*.

[70] Lu J, Lobarinas E, Deng A (2011). GABAergic neural activity involved in salicylate-induced auditory cortex gain enhancement. *Neuroscience*.

[71] Ralli M, Lobarinas E, Fetoni AR, Stolzberg D, Paludetti G, Salvi R (2010). Comparison of salicylate- and quinine-induced tinnitus in rats: development, time course, and evaluation of audiologic correlates. *Otology and Neurotology*.

[72] Paxinos G, Watson C (2004). *The Rat Brain in Stereotaxic Coordinates*.

[73] Stebbins WC, Miller JM (1964). Reaction time as a function of stimulus intensity for the monkey. *Journal of the Experimental Analysis of Behavior*.

[74] Lauer AM, Dooling RJ (2007). Evidence of hyperacusis in canaries with permanent hereditary high-frequency hearing loss. *Seminars in Hearing*.

[75] May BJ, Little N, Saylor S (2009). Loudness perception in the domestic cat: reaction time estimates of equal loudness contours and recruitment effects. *Journal of the Association for Research in Otolaryngology*.

[76] Bordi F, LeDoux J (1992). Sensory tuning beyond the sensory system: an initial analysis of auditory response properties of neurons in the lateral amygdaloid nucleus and overlying areas of the striatum. *The Journal of Neuroscience*.

[77] Jastreboff PJ, Jastreboff MM (2003). Tinnitus retraining therapy for patients with tinnitus and decreased sound tolerance. *Otolaryngologic Clinics of North America*.

[78] Vanneste S, Van de Heyning P, De Ridder D (2011). Contralateral parahippocampal gamma-band activity determines noise-like tinnitus laterality: a region of interest analysis. *Neuroscience*.

[79] Lockwood AH, Salvi RJ, Coad ML, Towsley ML, Wack DS, Murphy BW (1998). The functional neuroanatomy of tinnitus: evidence for limbic system links and neural plasticity. *Neurology*.

[80] Vanneste S, van Dongen M, De Vree B (2013). Does enriched acoustic environment in humans abolish chronic tinnitus clinically and electrophysiologically? A double blind placebo controlled study. *Hearing Research*.

[81] Vanneste S, De Ridder D (2013). Differences between a single session and repeated sessions of 1 Hz TMS by double-cone coil prefrontal stimulation for the improvement of tinnitus. *Brain Stimulation*.

[82] Brien J-A (1993). Ototoxicity associated with salicylates. A brief review. *Drug Safety*.

[83] Brennan JF, Brown CA, Jastreboff PJ (1996). Salicylate-induced changes in auditory thresholds of adolescent and adult rats. *Developmental Psychobiology*.

[84] Dauman R, Bouscau-Faure F (2005). Assessment and amelioration of hyperacusis in tinnitus patients. *Acta Oto-Laryngologica*.

[85] Coelho CB, Sanchez TG, Tyler RS (2007). Hyperacusis, sound annoyance, and loudness hypersensitivity in children. *Progress in Brain Research*.

[86] Stolzberg D, Chrostowski M, Salvi RJ, Allman BL (2012). Intracortical circuits amplify sound-evoked activity in primary auditory cortex following systemic injection of salicylate in the rat. *Journal of Neurophysiology*.

[87] Campeau S, Davis M (1995). Involvement of the central nucleus and basolateral complex of the amygdala in fear conditioning measured with fear-potentiated startle in rats trained concurrently with auditory and visual conditioned stimuli. *The Journal of Neuroscience*.

[88] Campeau S, Davis M (1995). Involvement of subcortical and cortical afferents to the lateral nucleus of the amygdala in fear conditioning measured with fear-potentiated startle in rats trained concurrently with auditory and visual conditioned stimuli. *The Journal of Neuroscience*.

[89] Swerdlow NR, Caine SB, Geyer MA (1992). Regionally selective effects of intracerebral dopamine infusion on sensorimotor gating of the startle reflex in rats. *Psychopharmacology*.

[90] Muller JM, Moore H, Myers MM, Shair HN (2008). Ventral striatum dopamine D2 receptor activity inhibits rat pups’ vocalization response to loss of maternal contact. *Behavioral Neuroscience*.

[91] Ciucci MR, Ahrens AM, Ma ST (2009). Reduction of dopamine synaptic activity: degradation of 50-kHz ultrasonic vocalization in rats. *Behavioral Neuroscience*.

[92] Jastreboff PJ, Sasaki CT (1994). An animal model of tinnitus: a decade of development. *American Journal of Otology*.

[93] Boettcher FA, Bancroft BR, Salvi RJ (1990). Concentration of salicylate in serum and perilymph of the Chinchilla. *Archives of Otolaryngology—Head and Neck Surgery*.

[94] Jastreboff PJ, Hansen R, Sasaki PG, Sasaki CT (1986). Differential uptake of salicylate in serum, cerebrospinal fluid, and perilymph. *Archives of Otolaryngology—Head and Neck Surgery*.

[95] Caperton KK, Thompson AM (2010). Activation of serotonergic neurons during salicylate-induced tinnitus. *Laryngoscope*.

[96] Wu JL, Chiu TW, Poon PWF (2003). Differential changes in Fos-immunoreactivity at the auditory brainstem after chronic injections of salicylate in rats. *Hearing Research*.

[97] Bordi F, LeDoux J, Clugnet MC, Pavlides C (1993). Single-unit activity in the lateral nucleus of the amygdala and overlying areas of the striatum in freely behaving rats: rates, discharge patterns, and responses to acoustic stimuli. *Behavioral Neuroscience*.

[98] Zou QZ, Shang XL (2012). Effect of salicylate on the large GABAergic neurons in the inferior colliculus of rats. *Acta Neurologica Belgica*.

[99] Yin S-H, Tang A-Z, Xing X-L, Tan S-H, Xie L-H (2006). Effects of sodium salicylate on the expressions of gamma-aminobutyricacid and glutamate and auditory response properties of the inferior colliculus neurons. *Sheng Li Xue Bao*.

[100] Jastreboff PJ, Brennan JF, Sasaki CT (1991). Quinine-induced tinnitus in rats. *Archives of Otolaryngology—Head and Neck Surgery*.

[101] Brennan JF, Jastreboff PJ (1991). Generalization of conditioned suppression during salicylate-induced phantom auditory perception in rats. *Acta Neurobiologiae Experimentalis*.

[102] Galiñanes GL, Braz BY, Murer MG (2011). Origin and properties of striatal local field potential responses to cortical stimulation: temporal regulation by fast inhibitory connections. *PLoS ONE*.

[103] McFadden D, Plattsmier HS, Pasanen EG (1984). Aspirin-induced hearing loss as a model of sensorineural hearing loss. *Hearing Research*.

[104] Happel MFK, Jeschke M, Ohl FW (2010). Spectral integration in primary auditory cortex attributable to temporally precise convergence of thalamocortical and intracortical input. *The Journal of Neuroscience*.

[105] Sokal DM, Giarola AS, Large CH (2005). Effects of GABAB, 5-HT1A, and 5-HT2 receptor stimulation on activation and inhibition of the rat lateral amygdala following medial geniculate nucleus stimulation in vivo. *Brain Research*.

[106] Sereda M, Hall DA, Bosnyak DJ (2011). Re-examining the relationship between audiometric profile and tinnitus pitch. *International Journal of Audiology*.

[107] Brozoski TJ, Bauer CA (2005). The effect of dorsal cochlear nucleus ablation on tinnitus in rats. *Hearing Research*.

[108] Kaltenbach JA, Afman CE (2000). Hyperactivity in the dorsal cochlear nucleus after intense sound exposure and its resemblance to tone-evoked activity: a physiological model for tinnitus. *Hearing Research*.

[109] Kaltenbach JA, Rachel JD, Alecia Mathog T, Zhang J, Falzarano PR, Lewandowski M (2002). Cisplatin-induced hyperactivity in the dorsal cochlear nucleus and its relation to outer hair cell loss: relevance to tinnitus. *Journal of Neurophysiology*.

[110] Mulders WHAM, Ding D, Salvi R, Robertson D (2011). Relationship between auditory thresholds, central spontaneous activity, and hair cell loss after acoustic trauma. *The Journal of Comparative Neurology*.

[111] Müller M, Klinke R, Arnold W, Oestreicher E (2003). Auditory nerve fibre responses to salicylate revisited. *Hearing Research*.

[112] Ma W-LD, Hidaka H, May BJ (2006). Spontaneous activity in the inferior colliculus of CBA/J mice after manipulations that induce tinnitus. *Hearing Research*.

[113] Wei L, Ding D, Sun W, Xu-Friedman MA, Salvi R (2010). Effects of sodium salicylate on spontaneous and evoked spike rate in the dorsal cochlear nucleus. *Hearing Research*.

[114] Zhang JS, Kaltenbach JA, Wang J, Kim SA (2003). Fos-like immunoreactivity in auditory and nonauditory brain structures of hamsters previously exposed to intense sound. *Experimental Brain Research*.

[115] Swerdlow NR, Geyer MA, Braff DL (2001). Neural circuit regulation of prepulse inhibition of startle in the rat: current knowledge and future challenges. *Psychopharmacology*.

[116] Li L, Fulton JD, Yeomans JS (1999). Effects of bilateral electrical stimulation of the ventral pallidum on acoustic startle. *Brain Research*.

[117] Mickley GA, Ferguson JL (1989). Enhanced acoustic startle responding in rats with radiation-induced hippocampal granule cell hypoplasia. *Experimental Brain Research*.

[118] Plourde G, Baribeau J, Bonhomme V (1997). Ketamine increases the amplitude of the 40-Hz auditory steady-state response in humans. *British Journal of Anaesthesia*.

